# Anti-Interleukin-6 Promotes Allogeneic Bone Marrow Engraftment and Prolonged Graft Survival in an Irradiation-Free Murine Transplant Model

**DOI:** 10.3389/fimmu.2017.00821

**Published:** 2017-07-19

**Authors:** Nicolas Granofszky, Andreas M. Farkas, Moritz Muckenhuber, Benedikt Mahr, Lukas Unger, Svenja Maschke, Nina Pilat, Raimund Holly, Mario Wiletel, Heinz Regele, Thomas Wekerle

**Affiliations:** ^1^Department of Surgery, Section of Transplant Immunology, Medical University of Vienna, Vienna, Austria; ^2^Clin. Institute of Pathology, Medical University of Vienna, Vienna, Austria

**Keywords:** transplantation, tolerance, chimerism, interleukin-6, regulatory T cells, bone marrow transplantation, co-stimulation blockade

## Abstract

Transfer of recipient regulatory T cells (Tregs) induces mixed chimerism and tolerance in an irradiation-free bone marrow (BM) transplantation (BMT) model involving short-course co-stimulation blockade and mTOR inhibition. Boosting endogenous Tregs pharmacologically *in vivo* would be an attractive alternative avoiding the current limitations of performing adoptive cell therapy in the routine clinical setting. Interleukin-6 (IL-6) potently inhibits Treg differentiation and its blockade was shown to increase Treg numbers *in vivo*. Therefore, we investigated whether IL-6 blockade can replace adoptive Treg transfer in irradiation-free allogeneic BMT. Treatment with anti-IL-6 instead of Treg transfer led to multi-lineage chimerism (persisting for ~12 weeks) in recipients of fully mismatched BM and significantly prolonged donor skin (MST 58 days) and heart (MST > 100 days) graft survival. Endogenous Foxp3^+^ Tregs expanded in anti-IL-6-treated BMT recipients, while dendritic cell (DC) activation and memory CD8^+^ T cell development were inhibited. Adding anti-IL-17 to anti-IL-6 treatment increased Treg frequencies, but did not further prolong donor skin graft survival significantly. These results demonstrate that IL-6 blockade promotes BM engraftment and donor graft survival in non-irradiated recipients and might provide an alternative to Treg cell therapy in the clinical setting.

## Introduction

Long-term allograft survival without the need of permanent immunosuppression remains an important goal in transplantation medicine (1). In this context, the co-transplantation of allogeneic bone marrow (BM) for the induction of mixed chimerism (i.e., co-existence of donor and recipient hematopoietic cells) evolved to be an attractive strategy (2). Several murine mixed chimerism protocols have been successfully translated to non-human primate (NHP) studies and several pilot trials performed in renal transplant patients demonstrate the clinical relevance of chimerism-based tolerance. However, the need of myelosuppressive pre-conditioning with its associated risks is a major challenge to more widespread clinical application (3–5).

Our group has recently developed a regimen free of myelosuppression that induces chimerism and long-term tolerance through a combination of donor BM transplantation (BMT) and recipient regulatory T cell (Treg) therapy under short-course CB (anti-CD40L and CTLA4-Ig) and of mTOR inhibition (6–9). Even though individualized cell therapy is a promising emerging therapeutic option (10, 11), its translation into clinical routine is impeded by numerous hurdles (12, 13). Hence, it is a desirable goal to find pharmacological alternatives that can be substituted for Treg cell therapy (14). Various promising protocols build on the principle of boosting thymically derived (t) Tregs *in vivo* (15) or the conversion of non-regulatory CD4^+^ T cells into peripherally derived (p) Tregs (16). With regard to non-cytotoxic BMT, our group could recently show that IL-2 complexes cannot replace adoptive Treg therapy (17) and thus alternative strategies need to be developed for this setting.

The pro-inflammatory cytokine interleukin-6 (IL-6) is a critical player in T cell and B cell (allo)immunity (18, 19). IL-6 is known for its versatile immunological functions, including its role as a key regulator of the Treg/Th17 balance where it prevents Treg development while promoting the differentiation of naïve T cells into Th17 cells (20, 21). IL-6 also promotes the activation and survival of effector T cells (22) and regulates *in vivo* dendritic cell (DC) differentiation through STAT3 (23). Moreover, it promotes T_FH_ lineage commitment, plasma cell progression, and high affinity antibody production (24, 25). Previously, it was shown that IL-6 deficiency can delay CD4-mediated cardiac allograft rejection (26–28). The role of IL-6 after allogeneic BMT is not fully understood yet, but it was shown that elevated serum levels correlate with acute graft-vs-host disease (GvHD) (29–31). Inhibition of the IL-6 signaling pathway reduces the severity of GvHD due to the augmentation of thymic-dependent and independent Treg reconstitution (32). Recently, we found that the co-transfer of high numbers of donor T cells triggers CB-resistant rejection of donor BM through IL-6-dependent bystander activation (33). Furthermore, disrupting the IL-6 signaling pathway with a humanized anti-IL-6 receptor (IL-6R) monoclonal antibody (tocilizumab) is approved for clinical use in Castleman’s disease, rheumatoid and juvenile arthritis (34–36). Notably, anti-IL-6R has been successfully tested as part of a delayed-tolerance-induction protocol in NHPs where long-term lung allograft survival was achieved *via* mixed chimerism with a non-myeloablative T cell depleting regimen (37). Moreover, tocilizumab revealed promising potential as GvHD prophylaxis after allogeneic stem-cell transplantation in a phase I/II trial (38). Several additional antibodies targeting the IL-6R or IL-6 itself are currently under clinical development (39–41).

To date, suitable protocols that achieve allogeneic BM engraftment without the need of cytoreductive therapy are still limited. The combination of CB, mTOR inhibition, and Treg therapy seems to be a promising approach. To increase clinical applicability, we investigated whether anti-IL-6 administration could obviate the need for Treg cell therapy in this non-cytotoxic murine mixed chimerism model.

## Materials and Methods

### Mice

Female C57BL/6 (B6, recipient, H-2^b^), BALB/c (donor, H-2^d^), and C3H/N (third party, H-2^k^) mice were purchased from Charles River Laboratories (Sulzfeld, Germany), housed under specific pathogen-free conditions, and used for experiments between 6 and 12 weeks of age. Congenic B6.SJL-Ptprca Pepcb/BoyJ (CD45.1^+^) and B10.D2 mice [donor, MHC mismatched, minor antigen (mHAg) matched to B6] were purchased from Jackson Laboratory (ME, USA) and bred in our own facility. Skin and heart transplantations were performed under controlled anesthesia with an intraperitoneally (i.p.) injected mixture of xylazin (5 mg/kg) and ketamine (100 mg/kg). All experiments were approved by the local review board of the Medical University of Vienna and the Austrian Federal Ministry of Science, Research and Economy and were performed in accordance with national and international guidelines of laboratory animal care (permission number GZ: BMWFW-66.009/0377-WF/V/3b/2016 and GZ: BMWFW-66.009/0303-WF/V/3b/2016).

### BM Transplantation and *In Vivo* Antibody Treatment Regimens

Groups of age-matched C57BL/6 recipients received 20 × 10^6^ unseparated BM cells or splenocytes [as donor-specific transfusion (DST) control] from BALB/c donors by injection into the tail vein together with CB consisting of anti-CD154 mAb (1 mg, d0, anti-CD40L, MR1, BioXcell), hCTLA4-Ig (0.5 mg, d2, abatacept, Bristol Myers Squibb), and a short-course of rapamycin (0.1 mg, d-1, d0, and d2, LC Laboratories) (6). Indicated recipients were additionally treated with an anti-IL-6 mAb (1 mg on days −1, 1, and 3 and then 0.1 mg every other day until day 13, MP5-20F3, BioXcell) (27, 28). Where indicated, groups of mice received in addition (0.5 mg on days −1, 1, and 3 and 0.1 mg every other day until day 13) anti-IL-6R (15A7) or anti-IL-17A (BZN035, Novartis Pharma AG) (42, 43).

### Skin and Heart Transplantation

Full thickness tail skin from BALB/c, B10.D2, or C3H/N mice was grafted to recipient flanks (lateral thorax wall) 5 weeks after BMT and visually inspected thereafter at short intervals. Grafts were considered to be rejected when less than 10% of the graft remained viable (6). Cervical heterotopic heart transplantation was performed 5 weeks after BMT, as described previously (44). Briefly, the recipient’s right external jugular vein and common carotid artery were everted over a cuff. The donor heart was harvested and flushed in a retrograde fashion with 4 mL HTK solution (Custodiol, Koehler Chemie, Alsbach-Haenlien, Germany) through the aortic arch. The pulmonary artery was connected with the external jugular vein and the aortic trunk with the common carotid artery. Heart allograft survival was determined by visual inspection and palpation at least twice weekly during long-term follow-up. End of graft survival was defined as complete cessation of heartbeat and was confirmed by histological analysis.

### Histology

Skin and heart grafts were harvested at the end of the experiment and fixed in 7.5% formalin. Paraffin blocks were sectioned and stained with hematoxylin and eosin (H&E) according to standard protocols. Histological slides were then scanned by Aperio ScanScope scanner (Aperio Technologies, Inc., Vista, CA, USA) and analyzed with ImageScope software. Additionally, grading of skin grafts was performed according to Banff 2007 working classification of skin-containing composite tissue allograft pathology and hearts were scored according to the International Society for Heart and Lung Transplantation (ISHLT) 2005 guidelines for cellular rejection by a pathologist blinded to the experimental background of samples.

### Mixed Lymphocyte Reaction (MLR) and Suppression Assay

Regarding MLR 5 × 10^5^ congenic C57BL/6 CD45.1^+^ responder cells (unseparated splenocytes) were cultured for 5 days in RPMI1640 supplemented with 10% FCS (Linaris), PenStrep (100 U Penicillin, 100 µg Streptomycin/ml; Sigma), 10 mM Hepes (MP Biomedicals), 1 mM Sodium Pyruvat (Sigma), and 1× non-essential amino acids (Sigma). Responder cells were stimulated with equal numbers of stimulator cells (unseparated splenocytes) from BALB/c (allogeneic) and C57BL/6 (self) or with Medium. Proliferation was measured by staining Ki-67 in responding (CD45.1^+^) CD4^+^ and CD8^+^ T cells after 5 days of culture. For the *in vitro* suppression assay, a 96-well plate was pre-coated overnight with 1 µg/ml anti-CD3 (145-2C11, BioXcell) diluted in PBS. On the next day, 4 × 10^5^ naïve responder CD45.1^+^ splenocytes (VPD 450 pre-labeled) were cocultured with indicated numbers of Tregs (for a responder:Treg ratio of 1:0, 1:1, 2:1, 4:1, 8:1, and 16:1) isolated on day 14 from BMT recipients (receiving CB and rapamycin) either with or without anti-IL-6. Tregs were purified by magnetic bead separation using the MACS CD4^+^CD25^+^ regulatory T-cell Isolation Kit (Miltenyi Biotec). Purity of separated population was >90%. After 4 days of incubation responder cell proliferation was assessed by measuring the dilution of VPD450. The percentage of suppression was calculated using the formula: [(% proliferation Tresp. cells alone − % of Tresp. cells treated with Treg)/% of Tresp. cells alone] × 100.

### Flowcytometric Analysis

Three-color flow cytometric analysis of multi-lineage chimerism was performed as described previously (45, 46). Briefly, chimerism was calculated as the net percentage of donor MHC class I^+^ [H2-D^d^-BIO (34-2-12)] in different leukocyte lineages {myeloid cells [Mac-1-FITC (M1/70)], B cells [CD19-PE (6D5)], CD4^+^ cells [CD4-APC-Cy7 (RM4-5)], and CD8^+^ cells [CD8-PE-Cy7 (53-6.7)]}. CD44-BIO (IM7), CD62L-PE (MEL-14), CD11c-BIO (N418), CD80-PERCP-Cy5 (16-10A1), CD86-FITC (GL-1), MHCII-PE-Cy7 (M5/114.15.2), CD40-PE (3/23), CD3-PerCP-Cy5 (17A2), SAV-APC, CD45.1-BIO (A20), CD45.2-BIO (104), and IgG-PE (Poly4053) were purchased from BioLegend (SanDiego, CA, USA). For intracellular staining, cells were permeabilized with the Foxp3/Transcription Factor Staining Buffer Set from eBioscience according to the manufacturer’s specification. Anti-mouse/rat Foxp3 APC (FJK-16s) and anti-mouse/rat Ki-67 PE-Cy7 (SolA15) were obtained from eBioscience. Flow cytometric analysis was performed with a BD FACS Canto II, and data were analyzed using FlowJo (10.0.8) software.

### Donor-Specific Antibodies (DSA)

5 × 10^5^ naïve recipient or donor thymocytes were incubated with 15 µl serum of indicated mice for 30 min at 37°C after heat inactivation of complement as described earlier (6). Briefly, binding of donor-reactive IgG to thymocytes was assessed by staining with anti-IgG (PE) and was measured by flow cytometry.

### Statistical Analysis

The statistical analyses were performed with SPSS 23.0 and GraphPad Prism 5.0 (GraphPad Software, Inc., La Jolla, CA, USA). Differences between chimerism levels were compared using ANOVA. Error Bars represent SD and statistics were performed with a two-sided Student’s *t*-test with equal variances. Skin and heart allograft survival was calculated according to the Kaplan–Meier product limit method and compared between groups using the log-rank test. Cardiac rejection scores were compared by using Fisher’s exact test. A *p*-value < 0.05 was considered statistically significant.

## Results

### Anti-IL-6 Promotes Allogeneic BM Engraftment and Prolongs Donor Skin Graft Survival

**Figure 1:**
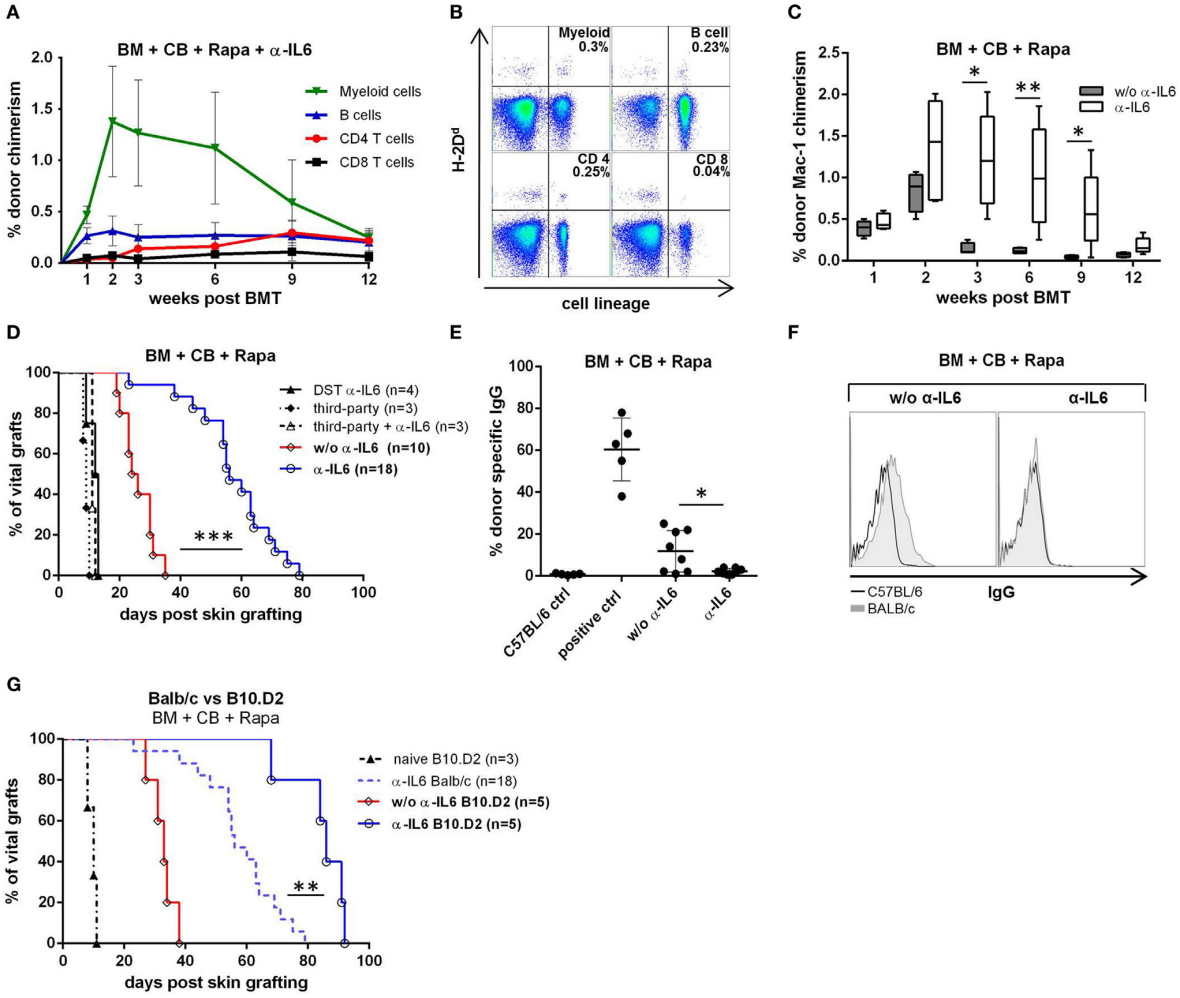
Enhanced bone marrow (BM) engraftment and prolonged skin graft survival with anti-IL-6. C57BL/6 recipients received fully mismatched BALB/c BM, co-stimulation blockade (CB), and rapamycin (Rapa). Additionally, indicated groups received anti-IL-6 mAb (α-IL6). Data shown in **(A,C,D)** are pooled from three independent experiments. **(A)** Mean percentage donor (H-2D^d^) chimerism among peripheral blood leukocyte lineages was followed over time in anti-IL-6 treated recipients (*n* = 18). **(B)** Two-color flow cytometry plots of lineage specific chimerism are shown for one representative anti-IL-6-treated recipient at week 12. **(C)** Percentage myeloid chimerism with and without anti-IL-6 is shown over time as box and whisker plots (*n* = 18 vs 10). **(D)** Survival of donor and third-party skin grafted 5 weeks post-BMT or splenocyte transfer [donor-specific transfusion (DST) α-IL-6] is depicted for the indicated groups. **(E)** IgG binding to BL/6 (C57BL/6 control, *n* = 5) or BALB/c (i.e., donor, with or w/o anti-IL-6, *n* = 8/grp) thymocytes was measured by flow cytometry 100 days post skin grafting. Serum from sensitized untreated BL/6 mice was used as positive control (*n* = 5). **(F)** Representative histograms of bound IgG are shown for anti-IL-6-treated or untreated mice. **(G)** Survival of B10.D2 skin grafted onto BALB/c BM recipients with or without anti-IL-6 is shown (*note*: the α-IL-6 Balb/c group is the same shown in panel D). Naïve B10.D2 control represents untreated BL/6 mice that received B10.D2 skin (*p ≤ 0.05, **p ≤ 0.01, ***p ≤ 0.001).

To investigate whether IL-6 blockade can replace Treg cell therapy in an irradiation-free BMT setting, C57BL/6 mice received fully mismatched BALB/c BM, CB (anti-CD40L mAb and CTLA4-Ig), rapamycin and in addition anti-IL-6 (instead of adoptive Treg transfer). All mice receiving anti-IL-6 developed early multi-lineage chimerism (within the myeloid and B cell lineages) that persisted for up to 12 weeks (18/18 chimeric at week 2, 6/18 mice remained chimeric at week 12, pooled results from three independent experiments). Noticeably, T cell chimerism appeared in most anti-IL-6 treated mice between weeks 6 and 9 and lasted until week 12 (Figures 1A,B), whereas no T cell chimerism was detectable in mice without anti-IL-6 (week 9: 14/18 with anti-IL-6 vs 0/10 without, *p* = 0.017). Among all measured leukocyte lineages, the myeloid population showed the highest chimerism levels over the whole period, peaking at week 2. In contrast, almost all control mice without anti-IL-6 (and without Treg transfer) lost myeloid (and B cell, data not shown) chimerism by week 3, or shortly thereafter (week 3: 1/10 chimeric vs 18/18 chimeric with anti-IL-6, *p* = 0.021) (Figure 1C), consistent with our previous experience (6).

To determine whether anti-IL-6 leads to donor-specific tolerance, donor skin was transplanted ~5 week’s post-BMT (i.e., 22 days after the last dose of anti-IL-6). Donor skin graft survival was significantly prolonged in anti-IL-6-treated mice [median survival time (MST) 58 days, *n* = 18] compared to control mice without anti-IL-6 (MST 24 days, *n* = 10, *p* = 0.0001). Third-party skin grafts were rapidly rejected in all groups (MST 11 and 10.5 days, respectively). To test whether the observed prolongation of BALB/c donor skin graft survival after BMT with anti-IL-6 depends on (transient) BM engraftment, a group of mice was treated with the same anti-IL-6 regimen but received donor splenocytes instead of BM (DST α-IL-6, *n* = 4). DST recipients promptly rejected donor skin (MST = 12.5 days), suggesting that BM engraftment is essential for graft prolongation and that DST does not suffice (Figure 1D). No DSA were detectable in sera of anti-IL-6 treated recipients 100 days post skin grafting, while more than half of mice without anti-IL-6 presented detectable levels of DSA (0/8 vs 5/8, *p* = 0.019), indicating that donor-specific humoral immunity is prevented in anti-IL-6 treated mice (Figures 1E,F).

Minor histocompatibility antigens (mHAg) are a barrier to tolerance induction through chimerism (47, 48). To investigate whether mHAg disparities are responsible for the late rejection of donor skin grafts in anti-IL-6-treated recipients, B10.D2 skin was grafted instead of BALB/c skin. Survival of B10.D2 grafts was significantly prolonged compared to BALB/c grafts (MST 86 vs 58 days, *p* = 0.0014) but all grafts were eventually rejected (Figure 1G), suggesting that mHAg disparities are not solely responsible for late rejection.

These results show that anti-IL-6 prolongs donor skin graft survival due to enhanced allogeneic BM engraftment.

### Anti-IL-6 Leads to Long-term Donor Heart Graft Acceptance

**Figure 2:**
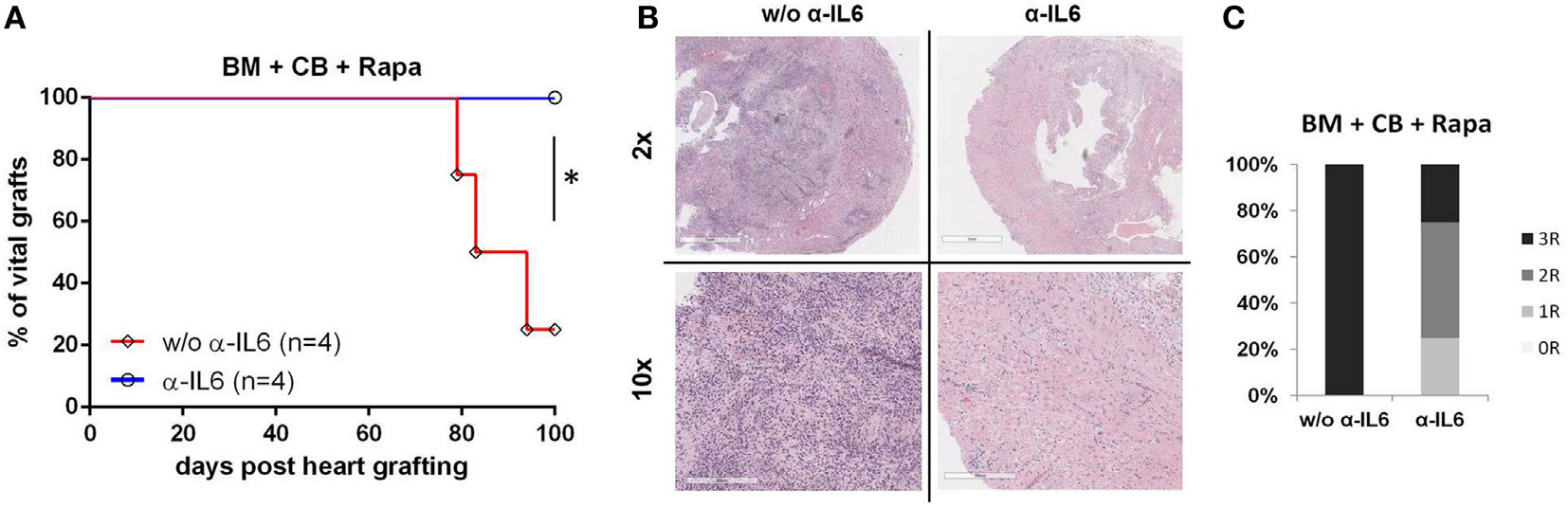
Long-term heart graft survival with anti-IL-6 BALB/c bone marrow (BM) recipients under CB, Rapa, and with or without anti-IL-6-received donor heart grafts 5 weeks post-BMT. Grafts were harvested 100 days post transplantation for histopathologic analysis. **(A)** Survival of donor hearts in BM transplantation recipients treated with or without anti-IL-6. **(B)** Representative H&E images of heart grafts [original magnifications 2× (top) and 10× (bottom)] are shown. **(C)** Clinical International Society for Heart and Lung Transplantation rejection score of donor hearts from both groups (*n* = 4/grp) (*p ≤ 0.05).

Skin grafts are generally considered to be the most stringent test for tolerance, while vascularized heart grafts are better suited to assess chronic rejection. Therefore, additional groups were transplanted with donor hearts 5 weeks post-BMT. Remarkably, in anti-IL-6-treated BMT recipients all donor hearts remained viable with a palpable heart beat for the length of the follow-up (*n* = 4, 100 days), whereas 3/4 recipients from the group without anti-IL-6 rejected their grafts (MST 88.5, *p* = 0.04) (Figure 2A). Histopathologic examination revealed lower rejection scores in mice treated with anti-IL-6 [median ISHLT score 2 (anti-IL-6) vs 4 (w/o anti-IL-6)] (Figure 2C). Heart grafts from mice without anti-IL-6 showed severe signs of cellular rejection, including extensive lymphocytic infiltrations, myocardial fibrosis, and necrosis, while three of four grafts from anti-IL-6 treatment demonstrated only mild to moderate signs of rejection, including lower rates of pericardial inflammation and intima arteritis (Figure 2B).

Hence, these data demonstrate that anti-IL-6 treatment leads to long-term acceptance of donor heart grafts in an irradiation-free BMT setting.

### IL-6 Blockade Induces Endogenous Treg Expansion and Proliferation after Allogeneic BMT

**Figure 3:**
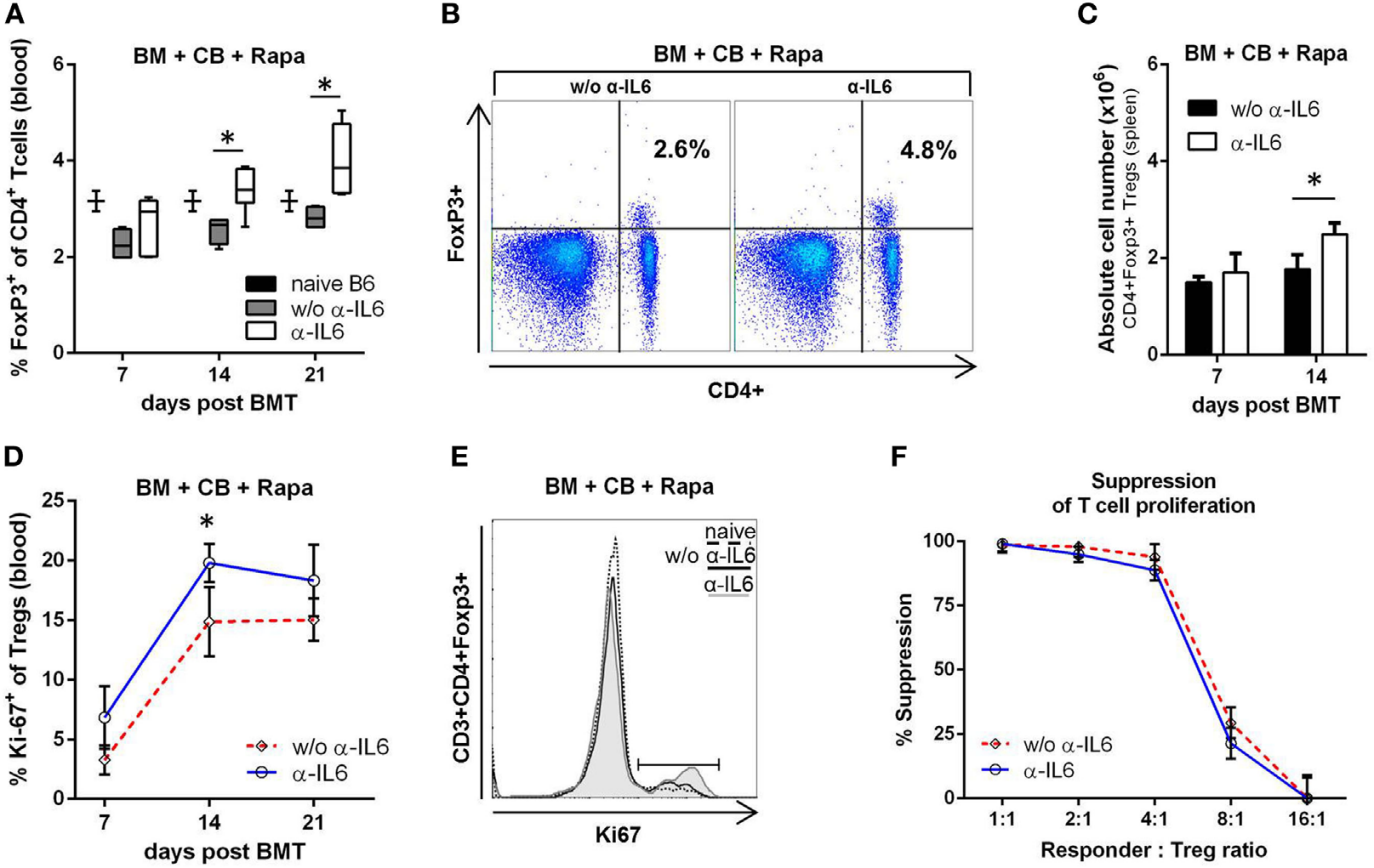
Anti-IL-6 therapy promotes regulatory T cell (Treg) expansion after BM transplantation (BMT). **(A)** Frequency of CD3^+^CD4^+^Foxp3^+^ Tregs was measured in blood in groups treated with anti-IL-6 *(n* = 18) or without anti-IL-6 (*n* = 10) on day 7, 14, and 21 post-BMT. Naïve B6 control represents untreated BL/6 mice (*n* = 3). **(B)** Two-color flow cytometry plots of representative mice at day 21 post-BMT are shown. **(C)** Absolute cell number of Tregs in the spleen is shown for both groups (*n* = 4/grp). **(D)** Proliferation of peripheral blood Tregs was determined by measuring intracellular expression of Ki-67 (anti-IL-6 *n* = 18 and without *n* = 10). **(E)** Representative histograms of Ki-67 expression are shown for day 14. **(F)** Percent suppression of polyclonally stimulated CD45.1^+^ responder splenocytes is shown for different doses of CD45.2^+^ Tregs isolated on day 14 from BMT recipients treated with or without anti-IL-6 (*n* = 3/grp) (*p ≤ 0.05).

Regulatory T cells modulate immune responsiveness to alloantigens, thereby modulating graft survival (49). To test whether the chimerism- and tolerance-promoting effects of anti-IL-6 administration are associated with *in vivo* boosting of endogenous Tregs, we measured the frequency, number, and proliferation of Foxp3^+^CD4^+^ T cells. Anti-IL-6 was associated with significantly increased frequencies of Foxp3 Tregs in blood (on days 14 and 21 post-BMT) (Figures 3A,B) and spleen (not shown), with an approximately 30% increase in absolute Treg numbers in spleen (Figure 3C). Moreover, Treg proliferation was elevated, as measured by the proliferation marker Ki-67 (Figures 3D,E). As it has been reported that CD4^+^CD25^+^ Tregs isolated from IL-6-deficient mice are more potent than those from WT mice (27), an *in vitro* suppression assay with polyclonally activated CD45.1^+^ splenocytes as responders and descending numbers of CD45.2^+^CD4^+^CD25^+^ Tregs isolated from BMT recipients treated either with or without anti-IL-6 was performed. Tregs from both groups suppressed the proliferation of responder T cells to a comparable degree in a dose-dependent manner (Figure 3F).

These data provide evidence that anti-IL-6 therapy leads to a quantitative expansion of endogenous Tregs, but does not qualitatively alter their suppressive potency.

### DC Activation and CD8^+^ Memory T Cell Generation Are Reduced after Anti-IL-6 Therapy

**Figure 4:**
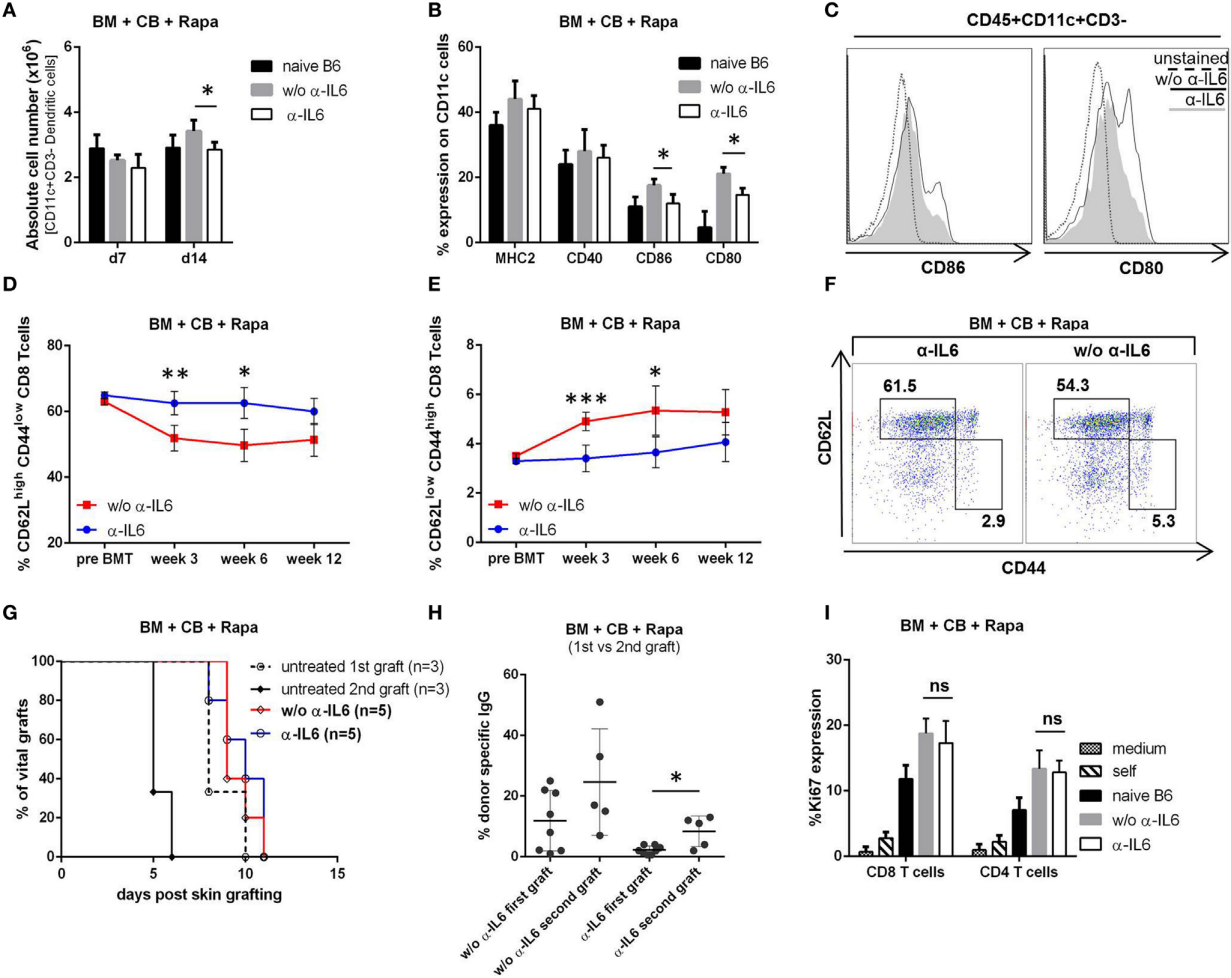
Anti-IL-6 reduces dendritic cell activation and CD8^+^ memory generation. **(A)** Absolute number of CD45^+^CD3^−^CD11c^+^ cells was determined in the spleen of groups treated with or without anti-IL-6 (*n* = 4/grp). Naïve B6 control represents untreated BL/6 mice (*n* = 2). **(B)** Percent expression of MHCII, CD40, CD80, and CD86 on CD11c^+^ cells on day 14 is shown. **(C)** Representative histograms of CD86 and CD80 expression on CD11c^+^ cells. **(D)** The percentage of CD62L^high^/CD44^low^ (naïve) and **(E)** CD62L^low^/CD44^high^ (effector memory) CD3^+^CD8^+^ blood T cells was followed over time in bone marrow (BM) recipients treated with anti-IL-6 (*n* = 10) or without (*n* = 5). **(F)** Two-color flow cytometry plots from representative mice are shown at week 3 post-BMT. **(G)** Survival of second donor skin grafts transplanted on the opposite flank 21 days after the rejection of a first donor graft is shown. Untreated B6 mice receiving either first or a second skin graft are shown as control. **(H)** Binding of serum IgG from mice treated with anti-IL-6 (first vs second graft) and without (first vs second graft) to BALB/c thymocytes was analyzed by flow cytometry (first *n* = 8/grp, second *n* = 5/grp). **(I)** Proliferation (Ki-67 expression in CD4^+^ and CD8^+^ T cells) was measured after 5 days of cell culture (*n* = 5/grp) in *in vitro* mixed lymphocyte reaction with responder splenocytes from BM recipient treated with or without anti-IL-6 (harvested after rejection of the last skin graft) stimulated with BALB/c splenocytes. Responder cells + medium (medium), responder cells + untreated BL/6 cells (self), and naïve BL/6 responder + BALB/c stimulators (naïve B6) served as controls (*p ≤ 0.05, **p ≤ 0.01, ***p ≤ 0.001).

Dendritic cells play a central role in alloimmunity through the presentation of alloantigens to naïve and memory T cells. IL-6 influences the maturation and activation of DCs (23). Therefore, recipient spleens of anti-IL-6-treated and untreated recipients were isolated on day 7 and 14 post-BMT and the DC population (CD45^+^CD11c^+^CD3^−^) was analyzed by flow cytometry. The number of DCs was significantly reduced in anti-IL-6-treated mice compared to the untreated group (~20% less at day 14, *p* = 0.018) (Figure 4A). Moreover, anti-IL-6-treated mice showed a significantly lower expression of CD80 and CD86 (anti-IL-6 CD86: 12%, CD80: 15% vs without anti-IL-6 CD86: 17% and CD80: 21%, *p* ≤ 0.05), suggesting reduced DC activation (MHCII and CD40 were similarly expressed in both groups) (Figures 4B,C).

Interleukin-6 is also important for the expansion and survival of effector T cells and the persistence of immunological memory (22). Thus, we followed the development of memory T cell subsets over time. In mice treated with anti-IL-6 the frequency of naïve CD8^+^ T cells (CD62L^high^/CD44^low^) remained constant at around 60% (similar to pre BMT levels), whereas it declined in mice not receiving anti-IL-6 (Figures 4D,F). In parallel, the frequency of CD8^+^ effector memory T cells (T_EM_, CD62L^low^/CD44^high^) remained relatively stable in the anti-IL-6 group and was significantly lower compared to the group without anti-IL-6 with the most pronounced difference at week 3 (~30% reduction, *p* ≤ 0.001) (Figures 4E,F). In contrast, generation of memory CD4^+^ T cells was not affected (data not shown). To investigate whether the tempered CD8^+^ T_EM_ development affects the “second-set” allograft response, a second donor skin graft was transplanted 3 weeks after the rejection of the first donor graft. Both groups rejected second donor skin grafts with similar kinetics as first grafts were rejected in untreated mice (Figure 4G). 2 weeks after second graft rejection low but detectable DSA levels appeared in anti-IL-6-treated mice (3/5), which were absent after rejection of the first graft (0/8, *p* = 0.039) (Figure 4H). In addition, an *in vitro* MLR with splenocytes, harvested from BM recipients after rejection of the first skin graft, revealed no significant difference regarding proliferation of both CD4^+^ and CD8^+^ T cells whether recipients were treated with anti-IL-6 or not, which is consistent with the *in vivo* results of skin graft rejection (Figure 4I).

Therefore, we conclude that anti-IL-6 reduces the expansion and activation of DCs and impairs CD8^+^ T_EM_ generation during BMT. However, this is not sufficient to delay rejection of a second skin graft.

### Adding Anti-IL-17 to Anti-IL-6 Therapy Further Elevates Treg Frequency but Does Not Extend Skin Graft Survival

Anti-IL-6 prolongs, but does not extend skin graft survival indefinitely in our model. Previously it has been demonstrated that IL-6 KO and IL-6R KO mice display phenotypic differences which might be explained by the fact that soluble IL-6R shares 60% homology with the IL-12p40 subunit which results in low-affinity interaction with IL-12 and IL-23 (50, 51). Furthermore, distinct outcomes were seen in a murine contact burn model where combinational treatment of anti-IL-6 and anti-IL-6R showed a significant reduction of the inflammatory response compared to anti-IL-6 or anti-IL-6R alone (52). Besides IL-6, increased serum IL-17 levels could be found in several murine acute and chronic allograft rejection models and it was shown that elevated IL-17 levels promote rejection in part by suppressing Treg expansion (53, 54). Thus, we asked whether the combination with anti-IL-17 or anti-IL-6R provides an additional benefit to anti-IL-6 treatment with regard to inducing long-term skin graft acceptance.

**Figure 5:**
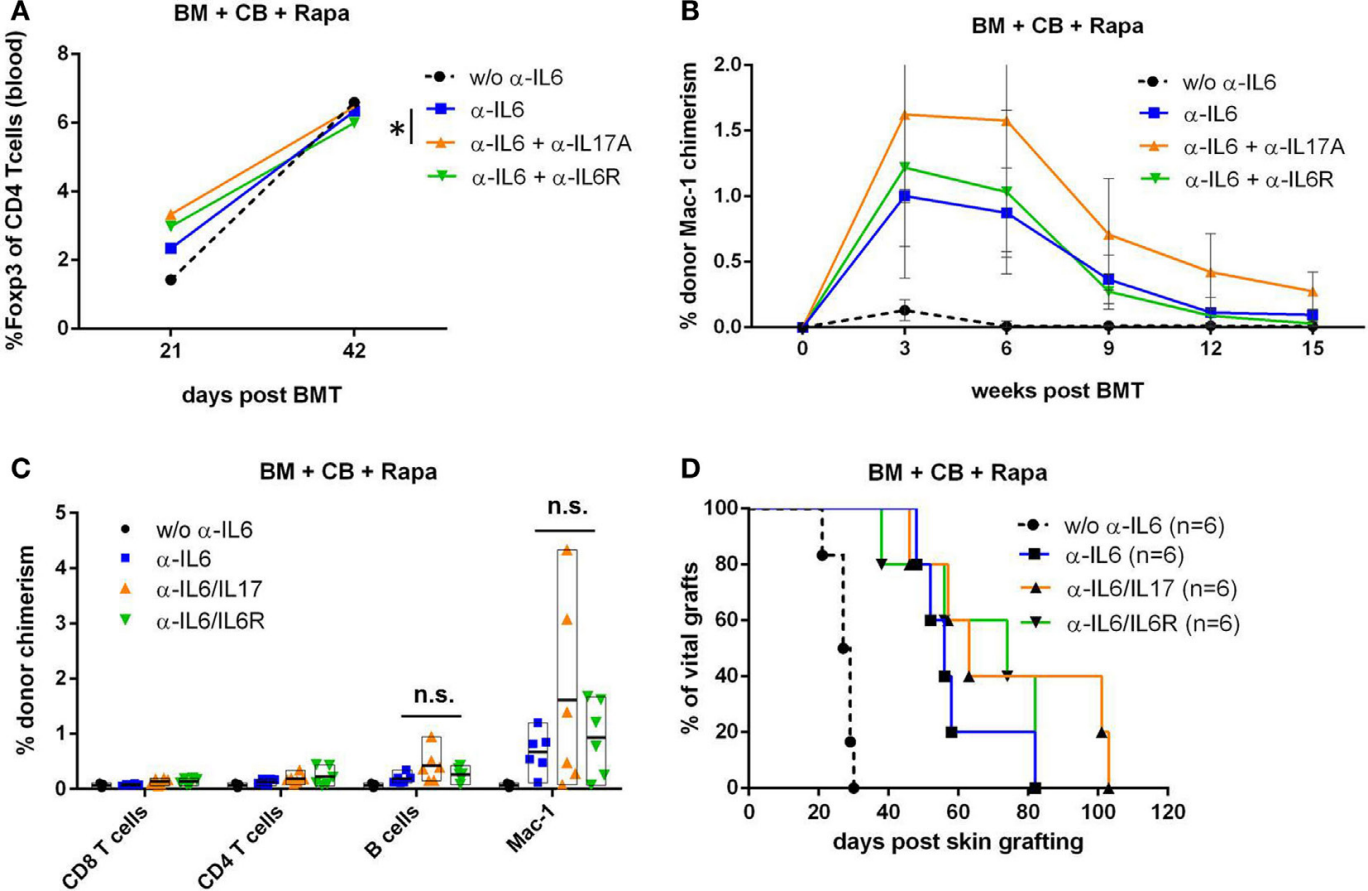
Combining anti-IL-17 with anti-IL-6 does not further extend skin graft survival. In an independent experiment, BALB/c bone marrow (BM) recipients (+CB+Rapa+anti-IL-6) were additionally treated with *in vivo* antibodies against IL-17A or IL-6 receptor and groups performed within this one experiment were compared to each other. **(A)** Mean percentage of blood CD3^+^CD4^+^Foxp3^+^ regulatory T cells were compared between indicated groups (*n* = 6/grp) 21 and 42 days post-BMT. **(B)** Mean percentage of donor Mac-1 chimerism in the indicated groups was followed over time. **(C)** Percentage of donor chimerism among indicated leukocyte lineages is shown for individual mice 42 days post-BMT. **(D)** Survival of donor skin is shown for BM transplantation (BMT) recipients treated with anti-IL-6 alone or in combination with anti-IL17 or anti-IL6R (*p ≤ 0.05).

Anti-IL-17 and anti-IL-6R further increased the percentage of blood Tregs on day 21 post-BMT when added to anti-IL-6 treatment (anti-IL-17: p* = 0.039 and anti-IL6R: *p* = 0.059; *n* = 6/grp) (Figure 5A). In contrast, anti-IL-17 alone showed no difference compared to anti-IL-6 (data not shown). Myeloid chimerism levels were numerically higher at all measured time points in mice treated with anti-IL-6 and anti-IL-17 compared to mice treated with anti-IL-6 alone (Figure 5B), with the clearest difference at week 6 (Figure 5C). There was, however, only a slight, non-significant prolongation of donor skin graft survival with addition of anti-IL-17 or anti-IL-6R (Figure 5D).

Taken together, these data suggest that the combination of anti-IL-6 and anti-IL-17 further expands endogenous blood Tregs and slightly improves BM engraftment, but is not sufficient to achieve permanent donor skin graft acceptance.

## Discussion

Although human pilot trials have shown promising results of tolerance induction *via* mixed chimerism (3, 5, 55), its widespread clinical translation is still a challenging process due to the need of myelosuppressive and cytotoxic recipient pre-conditioning. So far, the engraftment of clinically realistic BM doses in fully mismatched strain combinations remains a considerable challenge without the use of irradiation or cytotoxic antibody/drug treatment (56–58). Targeting pro-apoptotic factors such as Bcl-2 (with ABT-737) and the combination of Treg therapy with BM transplantation are the most effective ways to induce mixed chimerism without cytoreductive conditioning in mice developed so far (6, 59). In the present study, we demonstrate that IL-6 blockade exhibits a synergistic effect with CB and rapamycin which can largely replace adoptive Treg therapy in a murine non-myelosuppressive chimerism-based tolerance model.

Our data show that blocking IL-6 in conjunction with CB and mTOR inhibition promotes BM engraftment leading to transient multi-lineage chimerism. Anti-IL-6 treatment substantially prolonged donor skin graft survival and led to permanent donor heart graft survival with well-preserved histology. Moreover, the development of DSA was prevented with anti-IL-6. Treg transfer as performed in previous studies led to donor multi-lineage chimerism peaking around month 2 with a graduate decline thereafter, whereas anti-IL-6 therapy also led to early multi-lineage chimerism but with an earlier peak around week 2. However, Treg therapy associated chimerism remained relatively stable for the length of follow-up (up to 7 months post-BMT) with higher levels at all measured time points (e.g., myeloid chimerism ~1.5% with anti-IL-6 vs ~5% with Treg therapy at week 2 post-BMT). Moreover, Treg transfer led to permanent donor skin (vs MST 58 with anti-IL-6) and heart graft survival (similar to anti-IL-6) (6, 8).

The pro-tolerogenic effect of anti-IL-6 occurred only in the context of (transient) BM engraftment, as no such effect was seen when DST with donor splenocytes instead of BM was given. Since heart grafts survived until the end of the observation period while skin grafts were gradually rejected (although both express the same donor MHC molecules), tissue-specific antigens could be one reason for this difference in outcomes. However, it cannot be ruled out that the heart allografts would have been rejected at later times due to different effector mechanisms accounting for rejection of distinct tissues. mHAg disparities have a profound role in organ transplantation (60) and our group recently demonstrated that they significantly reduce the success rate of a CB-based chimerism protocol in inducing donor-specific tolerance (48). mHAg-matched donor skin showed prolonged survival but was not accepted indefinitely. Thus, mHAg disparities are not the only factor limiting donor skin graft survival under anti-IL-6 treatment and it is possible that the rejection of skin allografts was due to incomplete tolerance induction under the protocol used.

We found that chimerism and extended donor graft survival were associated with a quantitative increase in endogenous Treg numbers. It has been reported that isolated CD4^+^CD25^+^ cells from IL-6 deficient mice showed a greater potency to suppress IFN-γ production than wild-type Tregs (27). However, we observed no difference in the suppressive capacity of Tregs isolated from BMT recipients after anti-IL-6 treatment. IL-6 was previously shown to prolong T cell survival *via* maintenance of Bcl-2 expression and Fas ligand downregulation (61, 62). In addition, it could be demonstrated that IL-6 is an essential mediator for antigen-specific T cell priming (22). Moreover IL-6 KO recipients receiving an allogeneic heart graft presented a significant reduction in CD8^+^ T_EM_ generation (27). In the line with this, our results show that IL-6 blockade reduces the maturation of naïve CD8^+^ T cells to T_EMs_. Additionally, proliferation and priming of antigen-specific CD8^+^ T cells can be suppressed by Tregs (63), thus the decreased population of CD8^+^ T_EMs_ might also be related to the increased Treg levels seen upon anti-IL-6 treatment in our model. However, this effect was not sufficient to measurably affect the survival of a second donor skin graft. IL-6 also regulates DC differentiation and maturation (23, 64). Circulating DCs express MHC class II and low levels of CD40, CD80 and CD86 (65). In contrast, following stimulus, migrating DCs lose their ability to capture Ags, upregulate co-stimulatory molecules and secrete pro-inflammatory cytokines which further increase the inflammatory response (66). Our data show that anti-IL-6 reduces the expansion and activation of DCs during allogeneic BMT.

In a murine contact burn model the treatment of anti-IL-6 together with anti-IL-6R indicated further benefits in the clearance of the inflammatory response (52). Thus, we hypothesized that a combination of both antibodies (anti-IL6/anti-IL-6R) might improve the efficacy described above. Although the addition of anti-IL-6R further elevated Treg frequencies, chimerism levels were not significantly different to anti-IL-6 treatment alone, neither was skin graft survival. Previous studies have also suggested a synergistic effect of IL-6 blockade with blockade of other pro-inflammatory cytokines (67, 68). Combining anti-IL-6 and anti-IL-17 led to significantly increased Treg levels and a trend regarding higher myeloid chimerism levels. However, permanent chimerism or donor skin graft acceptance was not achieved, suggesting that other yet to be defined mechanisms need to be targeted in anti-IL-6 treated recipients.

Collectively, we conclude that anti-IL-6 therapy leads to BM engraftment and transient chimerism, with associated prolonged donor skin graft acceptance and indefinite donor heart survival. Thus, targeting IL-6 in combination with CB and mTOR inhibition represents a promising strategy for use in chimerism-based tolerance protocols.

## Ethics Statement

All experiments were approved by the local review board of the Medical University of Vienna and the Austrian Federal Ministry of Science, Research and Economy and were performed in accordance with national and international guidelines of laboratory animal care (permission number GZ: BMWFW-66.009/0377-WF/V/3b/2016 and GZ: BMWFW-66.009/0303-WF/V/3b/2016).

## Author Contributions

All authors have actively contributed to the study. NG and TW designed all experiments, analyzed and interpreted the data, and wrote the manuscript. NG, MM, BM, NP, AF, LU, SM, RH, and MW performed experiments. HR performed histopathological staining and analysis. All authors approved the final version of the manuscript.

## Conflict of Interest Statement

The author declares that the complete study was conducted in the absence of any commercial or financial relationship that could be construed as a potential conflict of interest.

## References

[1] Kawai T Leventhal J Madsen JC Strober S Turka LA Wood KJ. Tolerance: one transplant for life. Transplantation (2014) 98(2):117–21. 10.1097/TP.0000000000000260 24926829 PMC4101034

[2] Pilat N Wekerle T. Transplantation tolerance through mixed chimerism. Nat Rev Nephrol (2010) 6(10):594–605. 10.1038/nrneph.2010.110 20808286

[3] Leventhal J Abecassis M Miller J Gallon L Ravindra K Tollerud DJ Chimerism and tolerance without GVHD or engraftment syndrome in HLA-mismatched combined kidney and hematopoietic stem cell transplantation. Sci Transl Med (2012) 4(124):124ra28 10.1126/scitranslmed.3003509 PMC3610325 22399264

[4] Kawai T Sachs DH Sykes M Cosimi AB HLA-mismatched renal transplantation without maintenance immunosuppression. N Engl J Med (2013) 368(19):1850–2. 10.1056/NEJMc1213779 23656665 PMC3760499

[5] Scandling JD Busque S Dejbakhsh-Jones S Benike C Millan MT Shizuru JA Tolerance and chimerism after renal and hematopoietic-cell transplantation. N Engl J Med (2008) 358(4):362–8. 10.1056/NEJMoa074191 18216356

[6] Pilat N Baranyi U Klaus C Jaeckel E Mpofu N Wrba F Treg-therapy allows mixed chimerism and transplantation tolerance without cytoreductive conditioning. Am J Transplant (2010) 10(4):751–62. 10.1111/j.1600-6143.2010.03018.x 20148810 PMC2856406

[7] Pilat N Klaus C Hock K Baranyi U Unger L Mahr B Polyclonal recipient nTregs are superior to donor or third-party Tregs in the induction of transplantation tolerance. J Immunol Res (2015) 2015(10):9 10.1155/2015/562935 PMC4530277 26273682

[8] Pilat N Farkas AM Mahr B Schwarz C Unger L Hock K T-regulatory cell treatment prevents chronic rejection of heart allografts in a murine mixed chimerism model. J Heart Lung Transplant (2014) 33(4):429–37. 10.1016/j.healun.2013.11.004 24468120 PMC3991417

[9] Pilat N Mahr B Unger L Hock K Schwarz C Farkas AM Incomplete clonal deletion as prerequisite for tissue-specific minor antigen tolerization. JCI Insight (2016) 1(7):e85911. 10.1172/jci.insight.85911 27699263 PMC5033814

[10] Schliesser U Streitz M Sawitzki B. Tregs: application for solid-organ transplantation. Curr Opin Organ Transplant (2012) 17(1):34–41. 10.1097/MOT.0b013e32834ee69f 22143395

[11] Tang Q Bluestone JA. Regulatory T-cell therapy in transplantation: moving to the clinic. Cold Spring Harb Perspect Med (2013) 3(11):a015552. 10.1101/cshperspect.a015552 24186492 PMC3808774

[12] Schneider CK Salmikangas P Jilma B Flamion B Todorova LR Paphitou A Challenges with advanced therapy medicinal products and how to meet them. Nat Rev Drug Discov (2010) 9(3):195–201. 10.1038/nrd3052 20190786

[13] Trzonkowski P Bacchetta R Battaglia M Berglund D Bohnenkamp HR ten Brinke A Hurdles in therapy with regulatory T cells. Sci Transl Med (2015) 7(304):304s18. 10.1126/scitranslmed.aaa7721 26355029

[14] von Boehmer H Daniel C. Therapeutic opportunities for manipulating T(Reg) cells in autoimmunity and cancer. Nat Rev Drug Discov (2013) 12(1):51–63. 10.1038/nrd3683 23274471

[15] Webster KE Walters S Kohler RE Mrkvan T Boyman O Surh CD In vivo expansion of T reg cells with IL-2-mAb complexes: induction of resistance to EAE and long-term acceptance of islet allografts without immunosuppression. J Exp Med (2009) 206(4):751–60. 10.1084/jem.20082824 19332874 PMC2715127

[16] Francis RS Feng G Tha-In T Lyons IS Wood KJ Bushell A. Induction of transplantation tolerance converts potential effector T cells into graft-protective regulatory T cells. Eur J Immunol (2011) 41(3):726–38. 10.1002/eji.201040509 21243638 PMC3175037

[17] Mahr B Unger L Hock K Pilat N Baranyi U Schwarz C IL-2/α-IL-2 complex treatment cannot be substituted for the adoptive transfer of regulatory T cells to promote bone marrow engraftment. PLoS One (2016) 11(1):e0146245 10.1371/journal.pone.0146245 26731275 PMC4701413

[18] Jordan SC Choi J Kim I Wu G Toyoda M Shin B Interleukin-6, a cytokine critical to mediation of inflammation, autoimmunity and allograft rejection: therapeutic implications of IL-6 receptor blockade. Transplantation (2017) 101(1):32–44. 10.1097/TP.0000000000001452 27547870

[19] Hunter CA Jones SA. IL-6 as a keystone cytokine in health and disease. Nat Immunol (2015) 16(5):448–57. 10.1038/ni.3153 25898198

[20] Kimura A Kishimoto T. IL-6: regulator of Treg/Th17 balance. Eur J Immunol (2010) 40(7):1830–5. 10.1002/eji.201040391 20583029

[21] Veldhoen M Hocking RJ Atkins CJ Locksley RM Stockinger B. TGFbeta in the context of an inflammatory cytokine milieu supports de novo differentiation of IL-17-producing T cells. Immunity (2006) 24(2):179–89. 10.1016/j.immuni.2006.01.001 16473830

[22] Rochman I Paul WE Ben-Sasson SZ. IL-6 increases primed cell expansion and survival. J Immunol (2005) 174(8):4761–7. 10.4049/jimmunol.174.8.4761 15814701

[23] Park SJ Nakagawa T Kitamura H Atsumi T Kamon H Sawa S IL-6 regulates in vivo dendritic cell differentiation through STAT3 activation. J Immunol (2004) 173(6):3844–54. 10.4049/jimmunol.173.6.3844 15356132

[24] Harker JA Lewis GM Mack L Zuniga EI. Late interleukin-6 escalates T follicular helper cell responses and controls a chronic viral infection. Science (2011) 334(6057):825–9. 10.1126/science.1208421 21960530 PMC3388900

[25] Moens L Tangye SG. Cytokine-mediated regulation of plasma cell generation: IL-21 takes center stage. Front Immunol (2014) 5:65. 10.3389/fimmu.2014.00065 24600453 PMC3927127

[26] Lei J He F Wu M Zheng X Chen X Chen Z. Administration of anti-interleukin-6 monoclonal antibody prolongs cardiac allograft survival. Transpl Int (2010) 23(12):1271–81. 10.1111/j.1432-2277.2010.01125.x 20646257

[27] Zhao X Boenisch O Yeung M Mfarrej B Yang S Turka LA Critical role of proinflammatory cytokine IL-6 in allograft rejection and tolerance. Am J Transplant (2012) 12(1):90–101. 10.1111/j.1600-6143.2011.03770.x 21992708

[28] Booth AJ Grabauskiene S Wood SC Lu G Burrell BE Bishop DK. IL-6 promotes cardiac graft rejection mediated by CD4+ cells. J Immunol (2011) 187(11):5764–71. 10.4049/jimmunol.1100766 22025555 PMC3221839

[29] Tawara I Koyama M Liu C Toubai T Thomas D Evers R Interleukin-6 modulates graft-versus-host responses after experimental allogeneic bone marrow transplantation. Clin Cancer Res (2011) 17(1):77–88. 10.1158/1078-0432.ccr-10-1198 21047980 PMC3058832

[30] Imamura M Han M Hashino S Kobayashi H Imai K Kobayashi M Effects of interleukin-6 on hematopoiesis in allogeneic and syngeneic bone marrow chimeras. Immunobiology (1994) 191(1):21–37. 10.1016/S0171-2985(11)80265-2 7806257

[31] Symington FW Symington BE Liu PY Viguet H Santhanam U Sehgal PB. The relationship of serum IL-6 levels to acute graft-versus-host disease and hepatorenal disease after human bone marrow transplantation. Transplantation (1992) 54(3):457–62. 10.1097/00007890-199209000-00014 1412727

[32] Chen X Das R Komorowski R Beres A Hessner MJ Mihara M Blockade of interleukin-6 signaling augments regulatory T-cell reconstitution and attenuates the severity of graft-versus-host disease. Blood (2009) 114(4):891–900. 10.1182/blood-2009-01-197178 19491393 PMC2716024

[33] Hock K Pilat N Baranyi U Mahr B Gattringer M Klaus C Donor CD4 T cells trigger costimulation blockade-resistant donor bone marrow rejection through bystander activation requiring IL-6. Am J Transplant (2014) 14(9):2011–22. 10.1111/ajt.12823 25100658

[34] Nishimoto N Kanakura Y Aozasa K Johkoh T Nakamura M Nakano S Humanized anti-interleukin-6 receptor antibody treatment of multicentric Castleman disease. Blood (2005) 106(8):2627–32. 10.1182/blood-2004-12-4602 15998837

[35] Thompson CA FDA approves tocilizumab to treat rheumatoid arthritis. Am J Health Syst Pharm (2010) 67(4):254 10.2146/news100012 20133526

[36] Yokota S Imagawa T Mori M Miyamae T Aihara Y Takei S Efficacy and safety of tocilizumab in patients with systemic-onset juvenile idiopathic arthritis: a randomised, double-blind, placebo-controlled, withdrawal phase III trial. Lancet (2008) 371(9617):998–1006. 10.1016/S0140-6736(08)60454-7 18358927

[37] Tonsho M Lee S Aoyama A Boskovic S Nadazdin O Capetta K Tolerance of lung allografts achieved in nonhuman primates via mixed hematopoietic chimerism. Am J Transplant (2015) 15(8):2231–9. 10.1111/ajt.13274 25904524 PMC4569127

[38] Kennedy GA Varelias A Vuckovic S Le Texier L Gartlan KH Zhang P Addition of interleukin-6 inhibition with tocilizumab to standard graft-versus-host disease prophylaxis after allogeneic stem-cell transplantation: a phase 1/2 trial. Lancet Oncol (2014) 15(13):1451–9. 10.1016/S1470-2045(14)71017-4 25456364

[39] Huizinga TW Fleischmann RM Jasson M Radin AR van Adelsberg J Fiore S Sarilumab, a fully human monoclonal antibody against IL-6Ralpha in patients with rheumatoid arthritis and an inadequate response to methotrexate: efficacy and safety results from the randomised SARIL-RA-MOBILITY part A trial. Ann Rheum Dis (2014) 73(9):1626–34. 10.1136/annrheumdis-2013-204405 24297381 PMC4145418

[40] Voorhees PM Manges RF Sonneveld P Jagannath S Somlo G Krishnan A A phase 2 multicentre study of siltuximab, an anti-interleukin-6 monoclonal antibody, in patients with relapsed or refractory multiple myeloma. Br J Haematol (2013) 161(3):357–66. 10.1111/bjh.12266 23432640 PMC5837861

[41] Shaw S Bourne T Meier C Carrington B Gelinas R Henry A Discovery and characterization of olokizumab: a humanized antibody targeting interleukin-6 and neutralizing gp130-signaling. MAbs (2014) 6(3):774–82. 10.4161/mabs.28612 24670876 PMC4011921

[42] Ferrer IR Liu D Pinelli DF Koehn BH Stempora LL Ford ML. CD40/CD154 blockade inhibits dendritic cell expression of inflammatory cytokines but not costimulatory molecules. J Immunol (2012) 189(9):4387–95. 10.4049/jimmunol.1201757 23002440 PMC3478479

[43] Rampersad RR Tarrant TK Vallanat CT Quintero-Matthews T Weeks MF Esserman DA Enhanced Th17-cell responses render CCR2-deficient mice more susceptible for autoimmune arthritis. PLoS One (2011) 6(10):4. 10.1371/journal.pone.0025833 21991368 PMC3186765

[44] Schwarz C Unger L Mahr B Aumayr K Regele H Farkas AM The immunosuppressive effect of CTLA4 immunoglobulin is dependent on regulatory T cells at low but not high doses. Am J Transplant (2016) 16(12):3404–15. 10.1111/ajt.13872 27184870

[45] Wekerle T Sayegh MH Hill J Zhao Y Chandraker A Swenson KG Extrathymic T cell deletion and allogeneic stem cell engraftment induced with costimulatory blockade is followed by central T cell tolerance. J Exp Med (1998) 187(12):2037–44. 10.1084/jem.187.12.2037 9625763 PMC2212372

[46] Blaha P Bigenzahn S Koporc Z Schmid M Langer F Selzer E The influence of immunosuppressive drugs on tolerance induction through bone marrow transplantation with costimulation blockade. Blood (2003) 101(7):2886–93. 10.1182/blood-2002-10-3014 12433677

[47] Cao TM Lo B Ranheim EA Grumet FC Shizuru JA. Variable hematopoietic graft rejection and graft-versus-host disease in MHC-matched strains of mice. Proc Natl Acad Sci U S A (2003) 100(20):11571–6. 10.1073/pnas.2035077100 14504392 PMC208799

[48] Bigenzahn S Pree I Klaus C Pilat N Mahr B Schwaiger E Minor antigen disparities impede induction of long lasting chimerism and tolerance through bone marrow transplantation with costimulation blockade. J Immunol Res (2016) 2016:8635721. 10.1155/2016/8635721 27872868 PMC5107841

[49] Wood KJ Sakaguchi S. Regulatory T cells in transplantation tolerance. Nat Rev Immunol (2003) 3(3):199–210. 10.1038/nri1027 12658268

[50] Gearing DP Cosman D Homology of the p40 subunit of natural killer cell stimulatory factor (NKSF) with the extracellular domain of the interleukin-6 receptor. Cell (1991) 66(1):9–10. 10.1016/0092-8674(91)90131-H 2070420

[51] Schuster B Kovaleva M Sun Y Regenhard P Matthews V Grotzinger J Signaling of human ciliary neurotrophic factor (CNTF) revisited. The interleukin-6 receptor can serve as an alpha-receptor for CTNF. J Biol Chem (2003) 278(11):9528–35. 10.1074/jbc.M210044200 12643274

[52] Pallua N Low JF von Heimburg D Pathogenic role of interleukin-6 in the development of sepsis. Part II: significance of anti-interleukin-6 and anti-soluble interleukin-6 receptor-alpha antibodies in a standardized murine contact burn model. Crit Care Med (2003) 31(5):1495–501. 10.1097/01.CCM.0000065725.80882.BD 12771624

[53] Yuan X Paez-Cortez J Schmitt-Knosalla I D’Addio F Mfarrej B Donnarumma M A novel role of CD4 Th17 cells in mediating cardiac allograft rejection and vasculopathy. J Exp Med (2008) 205(13):3133–44. 10.1084/jem.20081937 19047438 PMC2605226

[54] Itoh S Kimura N Axtell RC Velotta JB Gong Y Wang X Interleukin-17 accelerates allograft rejection by suppressing regulatory T cell expansion. Circulation (2011) 124(11 Suppl):S187–96. 10.1161/circulationaha.110.014852 21911812

[55] Kawai T Cosimi AB Spitzer TR Tolkoff-Rubin N Suthanthiran M Saidman SL HLA-mismatched renal transplantation without maintenance immunosuppression. N Engl J Med (2008) 358(4):353–61. 10.1056/NEJMoa071074 18216355 PMC2819046

[56] Wekerle T Kurtz J Ito H Ronquillo JV Dong V Zhao G Allogeneic bone marrow transplantation with co-stimulatory blockade induces macrochimerism and tolerance without cytoreductive host treatment. Nat Med (2000) 6(4):464–9. 10.1038/74731 10742157

[57] Westerhuis G Maas WG Willemze R Toes RE Fibbe WE. Long-term mixed chimerism after immunologic conditioning and MHC-mismatched stem-cell transplantation is dependent on NK-cell tolerance. Blood (2005) 106(6):2215–20. 10.1182/blood-2005-04-1391 15928035

[58] Sykes M Szot GL Swenson KA Pearson DA. Induction of high levels of allogeneic hematopoietic reconstitution and donor-specific tolerance without myelosuppressive conditioning. Nat Med (1997) 3(7):783–7. 10.1038/nm0797-783 9212108

[59] Cippa PE Gabriel SS Chen J Bardwell PD Bushell A Guimezanes A Targeting apoptosis to induce stable mixed hematopoietic chimerism and long-term allograft survival without myelosuppressive conditioning in mice. Blood (2013) 122(9):1669–77. 10.1182/blood-2012-09-453944 23869083

[60] Dierselhuis M Goulmy E. The relevance of minor histocompatibility antigens in solid organ transplantation. Curr Opin Organ Transplant (2009) 14(4):419–25. 10.1097/MOT.0b013e32832d399c 19444105

[61] Ayroldi E Zollo O Cannarile L D’Adamio F Grohmann U Delfino DV Interleukin-6 (IL-6) prevents activation-induced cell death: IL-2-independent inhibition of Fas/fasL expression and cell death. Blood (1998) 92(11):4212–9. 9834226

[62] Teague TK Marrack P Kappler JW Vella AT. IL-6 rescues resting mouse T cells from apoptosis. J Immunol (1997) 158(12):5791–6. 9190930

[63] Chappert P Leboeuf M Rameau P Lalfer M Desbois S Liblau RS Antigen-specific Treg impair CD8(+) T-cell priming by blocking early T-cell expansion. Eur J Immunol (2010) 40(2):339–50. 10.1002/eji.200839107 19877007

[64] Jonuleit H Kuhn U Muller G Steinbrink K Paragnik L Schmitt E Pro-inflammatory cytokines and prostaglandins induce maturation of potent immunostimulatory dendritic cells under fetal calf serum-free conditions. Eur J Immunol (1997) 27(12):3135–42. 10.1002/eji.1830271209 9464798

[65] Kohrgruber N Halanek N Groger M Winter D Rappersberger K Schmitt-Egenolf M Survival, maturation, and function of CD11c- and CD11c+ peripheral blood dendritic cells are differentially regulated by cytokines. J Immunol (1999) 163(6):3250–9. 10477594

[66] Hancock WW Sayegh MH Zheng XG Peach R Linsley PS Turka LA. Costimulatory function and expression of CD40 ligand, CD80, and CD86 in vascularized murine cardiac allograft rejection. Proc Natl Acad Sci U S A (1996) 93(24):13967–72. 10.1073/pnas.93.24.13967 8943044 PMC19478

[67] Chen L Ahmed E Wang T Wang Y Ochando J Chong AS TLR signals promote IL-6/IL-17-dependent transplant rejection. J Immunol (2009) 182(10):6217–25. 10.4049/jimmunol.0803842 19414775 PMC2834528

[68] Shen H Goldstein DR. IL-6 and TNF-alpha synergistically inhibit allograft acceptance. J Am Soc Nephrol (2009) 20(5):1032–40. 10.1681/ASN.2008070778 19357252 PMC2678042

